# Perceived match between own and observed models’ bodies: influence of face, viewpoints, and body size

**DOI:** 10.1038/s41598-020-70856-8

**Published:** 2020-08-19

**Authors:** Lize De Coster, Pablo Sánchez-Herrero, Carlos Aliaga, Miguel A. Otaduy, Jorge López-Moreno, Ana Tajadura-Jiménez

**Affiliations:** 1 DEI Interactive Systems Group, Department of Computer Science and Engineering, Universidad Carlos III de Madrid, Avenida de la Universidad 30, 28911 Leganés, Madrid Spain; 2 Seddi Labs, Madrid, Spain; 3 Modeling and Virtual Reality Group, Department of Computer Science, Universidad Rey Juan Carlos, Madrid, Spain

**Keywords:** Psychology, Human behaviour

## Abstract

People are generally unable to accurately determine their own body measurements and to translate this knowledge to identifying a model/avatar that best represents their own body. This inability has not only been related to health problems (e.g. anorexia nervosa), but has important practical implications as well (e.g. online retail). Here we aimed to investigate the influence of three basic visual features—face presence, amount of viewpoints, and observed model size—on the perceived match between own and observed models’ bodies and on attitudes towards these models. Models were real-life models (Experiment 1) or avatar models based on participants’ own bodies (Experiment 2). Results in both experiments showed a strong effect of model size, irrespective of participants’ own body measurements. When models were randomly presented one by one, participants gave significantly higher ratings to smaller- compared to bigger-sized models. The reverse was true, however, when participants observed and compared models freely, suggesting that the mode of presentation affected participants’ judgments. Limited evidence was found for an effect of facial presence or amount of viewpoints. These results add evidence to research on visual features affecting the ability to match observed bodies with own body image, which has biological, clinical, and practical implications.

## Introduction

While our own body is one of the objects we are perceptually most familiar and experienced with, research indicates that the perception of our body is largely inaccurate. It has been suggested, for example, that people perceive a considerable mismatch between their actual body size/shape and their body image (the way we perceive our body irrespective of what the body actually looks like^1^)^2–7^. These distorted self-representations have been shown to be related to serious clinical disorders, such as body dysmorphia and anorexia nervosa^8,9^. Furthermore, this inability to accurately determine own body measurements has several other practical implications. Online retail experiences, for example, present users with scenarios that demand the ability to match one’s own body image with visual bodily information in order to make decisions on body and apparel fit. During such online shopping experiences, individuals usually observe the apparel on a model, with or without the option to choose the size and shape of the model they would like to use for this experience (virtual try-on^10^). Research indicates that using a model that represents the self (model self-congruity) increases the impact of virtual try-ons (e.g. more confidence in apparel fit, greater purchase intentions), and that websites and companies providing these experiences should focus on maximizing the perceived resemblance between the consumer and the model^11^. However, as mentioned above, there is a considerable mismatch between subjects’ perceived body measurements and their body image^2–7^, while other studies have observed that idealized avatars are generally preferred to avatars that are more truthful^12^. The question arises whether this incongruency—where customers are unable to identify and/or don’t prefer models that best represent their own body, even though such models are essential to an effective online shopping experience that is satisfying to both customers and retailers—contributes to the general dissatisfaction with online purchases and associated return rates that substantially limit online retail’s profitability^13–15^, and how this can be resolved.

In the current paper, we aimed to explore whether there are easily identifiable visual features that influence people’s ability to determine the perceived match between own and others’ bodies. To this end, we describe two all-female experiments that manipulated the presence of facial features, multiple viewpoints, and multiple body sizes to investigate the influence of these features in accurately determining the model that best represents a person’s own body measurements and general attitudes towards these models in a specific ecological context (online retail). To study how participants were able to match their internal body image with visual bodily information, we asked participants to make decisions on body and apparel fit, while observing real-life models (Experiment 1) or personalized avatar models based on a scan of participants’ own bodies (Experiment 2).

**Figure 1:**
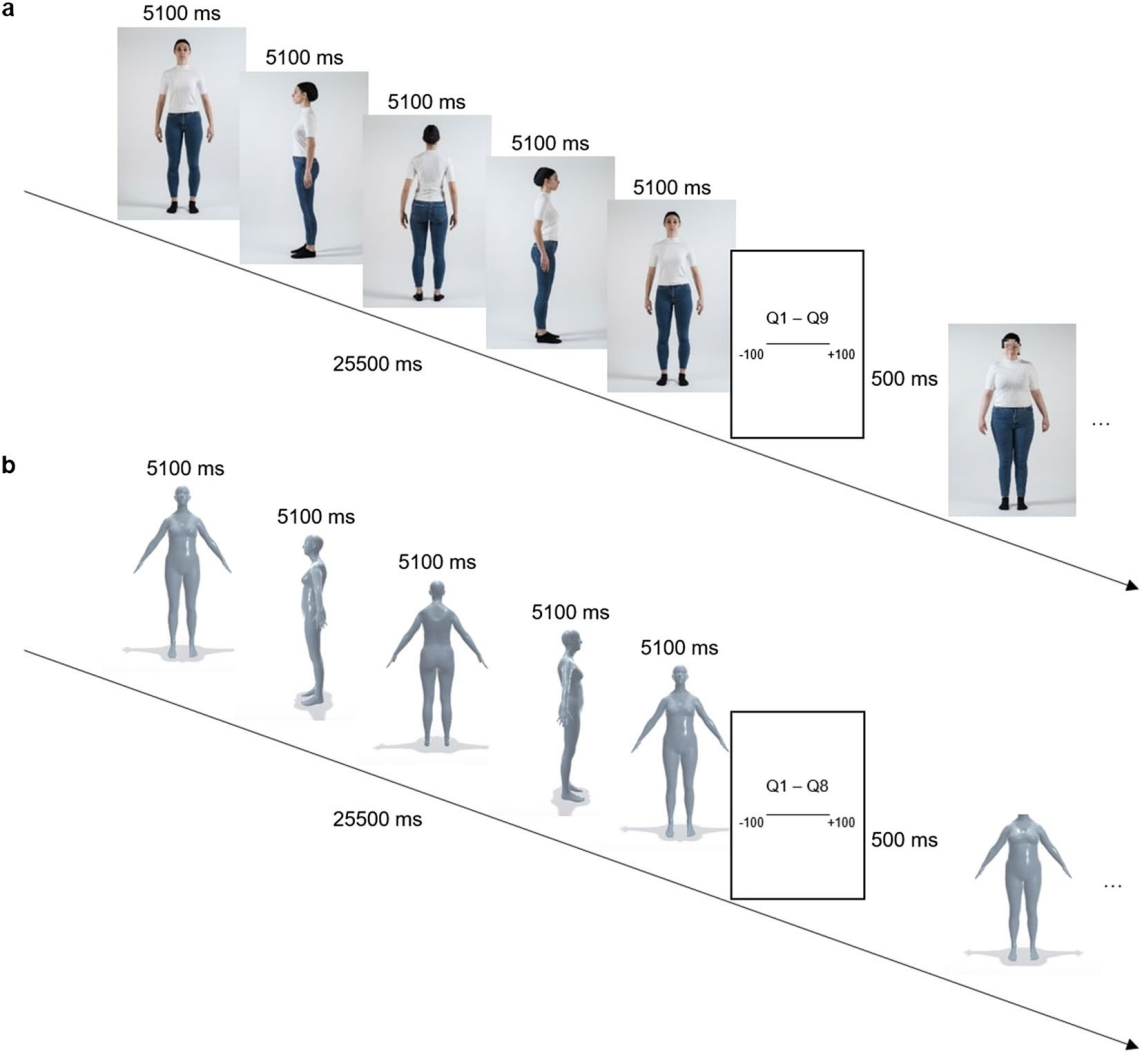
Procedure overview. Schematic overview of the procedure in (**a**) Experiment 1 and (**b**) Experiment 2, depicting a trial where one of the models is shown from four different viewpoints, with facial features. The subsequent trial depicts a model without facial features (face pixelation in Experiment 1 and removal of the face in Experiment 2). Q1–Q9 = nine experiment questions in Experiment 1. Q1–Q8 = eight experiment questions in Experiment 2.

In a first experiment, participants were presented with images of real-life models with different body sizes, dressed accordingly in various sizes of identical jeans and t-shirt. Participants observed these models from different amounts of viewpoints, as well as with or without recognizable facial features (see Fig. 1a), which resulted in three factors being manipulated: Face, View, and Model (size). Self-report, including questions concerning apparel fit, measurement correspondence, attractiveness, trust, and rebrowse potential (the desire to re-use the model for online shopping), was used. Furthermore, for questions that could be mapped to an accurate response (e.g. *How likely do you it is that this model’s measurements correspond to your own?*), participants were additionally asked to indicate the level of confidence in their response. Explicit confidence/certainty judgments, which fall within the field of metacognition, allow to assess the reliability of perception across different decisions and have been shown to relate to subjective rather than objective accuracy^16^. Based on previous literature and our experimental setup, we formulated the following set of hypotheses for the first experiment:

*Hypotheses related to Face*: A key feature in someone’s physical appearance is his/her face, being one of the most important factors in developing and maintaining self-identity^15,17,18^. Furthermore, humans are exceptionally adept at identifying specific individual faces in a short amount of time^19^. The importance of face identification skills is highlighted by their presence in early development^19^, the wide range of social difficulties encountered when these skills are compromised^20,21^, and specialized perceptual processes and neural mechanisms by face-selective regions in the cortex^22–24^. Further evidence for the importance of facial features is provided by research that has shown that another human’s face is a possible distracting attribute during social interactions that seems to capture observers’ attention the majority of the time^25^, and that this might be increased during video compared to live interactions^26^. Other research, on the other hand, found that observing another person’s face in a learning situation (where the other person had to demonstrate a particular task to participants) was beneficial to learning outcomes^27^. This raises the question whether the presence of facial features is helpful or distracting for identifying a self-congruent model. A second research question is whether facial features induce positive or negative attitudes towards observed models. Interestingly, some studies have suggested that abstract or absent facial features facilitate self-identification (given the contrast to photo-realistic faces that are easy to identify as someone else^28,29^), while others indicated that body and face independently predict perceived attractiveness of a female body^30^. Thus, given that research has shown that facial features have the potential to distract observers^25,26^ and since determining model self-congruency did not depend on the models’ faces, we expected models without a face to lead to more accurate identification of fit and measurement correspondence. However, we conversely expected ratings of trust in the model (personality) to be higher for models with a face given the importance of facial features during social interactions^31^. A ‘face’ condition was contrasted with a ‘no face’ condition by pixelating the face of the models in the latter.

*Hypotheses related to View*: Research on bodily processing has suggested that information about the whole body (rather than singular body parts) is essential for the configuration of mental body representations of both own and others’ bodies^32–34^. Others indicated, however, that despite the abundance of perceptual bodily information that is available, there are striking distortions in people’s perceptions of the relative proportions of their own and others’ bodies^35^. Furthermore, it has been suggested that there is a body-specific overestimation of several body parts belonging to participants’ own bodies, irrespective of whether participants had access to perceptual information about their body or not^5^. Thus, while it seems that having sufficient information facilitates accurate body perception and representation, it is unclear whether there is a limit to how much information is necessary and/or helpful in this process. Applied to online shopping contexts, it has not been systematically investigated whether and to which extent the possibility of viewing an avatar from multiple angles (i.e. providing more perceptual information, something which is often included in virtual try-on experiences) influences body size estimation of a perceived avatar and its self-congruency, as well as attitudes towards this avatar. Whereas results are contradictory as to whether the viewpoint from which a female body (significant advantage for three-quarter view^36^) or personalized avatar (no advantage for a specific viewpoint^37^) is perceived influences body size estimation, participants in previous studies were only able to view the avatar from one specific viewpoint. The aforementioned research indicates that—while sufficient visual information is paramount—more might not necessarily mean better. Research on more abstract information processing in online environments has shown that excessive information load is not beneficial to decision making processes^38,39^. It has been shown, for example, that information that overwhelms customers’ capacity for decision making results in consumers leaving an online store^40,41^. Additionally, it has been observed that the degree of information load and novelty (e.g. product attributes, brand alternatives) exhibits an inverted U-shaped relationship with intention to buy and explore respectively. Furthermore, information complexity seems to exert a negative influence on these intentions^42^. Based on this research, we expected that being able to observe a model and its apparel from a larger amount of viewpoints/angles would increase positive attitudes towards the model and lead to more accurate decisions regarding apparel and measurement fit. However, since research has shown that abundant (visual) information is not necessarily helpful or beneficial for decision making processes (within an online shopping context)^38–42^ or for body representation^32–34^, we hypothesized that a restricted number of viewpoints (e.g. eight) would be better than a full 360° rotation. Furthermore, we predicted that eight viewpoints (allowing for a three-quarter view^36^) would be better than one or four viewpoints.

*Hypotheses related to Model*: As described above, there seems to be a considerable mismatch between individuals’ actual body size/shape and the size/shape of their represented body (i.e. their body image). Research has shown that both over^43^-, and underestimation^44–49^ of own and others’ body size are found in adult and child populations. We predicted that such distortions would also be present in the current study, where participants had to make judgments (apparel fit, measurement correspondence, response confidence) based on a perceived match between their own body size and that of an observed model. Furthermore, due to a globally widespread body weight stigma^50^ that is particularly pervasive in women^51^, we hypothesized that attractiveness and rebrowse ratings would be higher for smaller-sized models, irrespective of participants’ own measurements.

In a second experiment, we wanted to replicate the findings of the first experiment with models that were based on participants’ own bodies, with the expectation that this would help participants better identify the model that best represented their own body and that such individualized avatar models would increase participants’ performance. Furthermore, we expected that they would show increased positive attitudes (e.g. trust) towards these models (similar to effects with self-avatars^52^). We created individual-specific avatars that were presented to participants with or without distortions in their body measurements (see Fig. 1b). We again investigated the influence of the presence of facial features, the number of viewpoints, and body measurements of the models observed on self-report of measurement correspondence (and confidence in this response), attractiveness, trust, body acceptance and identification^53,54^, and rebrowse potential. Our hypotheses were similar to the ones described in Experiment 1 for each factor. Regarding the effect of facial features, we contrasted models with a face (a generic face with recognizable facial features) with models whose face was completely removed. Inspired by current practices in online apparel retail, the latter was used to more clearly distinguish between both face conditions when observing an avatar whose facial features are less detailed than a real-life model.

In sum, our aim with both studies was to gain insights into the factors that contribute to the perceived match between participants’ own body image and bodies of observed models/avatars, as well as attitudes towards these observed bodies. Furthermore, by applying this research to a specific ecological context (online shopping), we aimed to provide a link with contemporary problems in online retail experiences.

## Results

### Experiment 1

#### Participant characteristics

Jeans size of participants was calculated using hips and waist circumvention measurements. After conversion using a standard European size chart, the distribution of jeans sizes among the 35 female participants was as follows: size 32 = 3, 34 = 7, 36 = 9, 38 = 6, 40 = 4, 42 = 3, 44 = 3 (representative of what would be expected in the female Spanish population^55^). Given gender differences in body evaluation^56^, only female participants were recruited.

A majority of the participants indicated to have bought an item online in the past year (27), of which 13 indicated to purchase online items equal to or more than once per week. 14 participants indicated never to have returned an item, while 13 participants had returned one or more items in the past year. The primary reason for returning the purchase was bad fit. See Table 1 for demographic and questionnaire data. When compared to a representative group of Spanish females^57,58^, participants’ scores seemed to fall within the normal ranges concerning general self-esteem and personality scores, although a relatively low score for the Agreeableness subscale of the Big 5 was observed in our participant group. Relatively higher scores were observed for Subjective importance of corporality and Behaviors oriented at maintaining shape of the Multidimensional Body-Self Relations Questionnaire (MBSRQ), while lower scores were observed for Self-evaluation of physical attractiveness and Care for physical aspect when compared to a large young Spanish female population^59^. However, normative scores for the MBSRQ are based on data from 2007, and it has been suggested that body dissatisfaction and body image concerns have changed over the years^60^.

**Table 1:** Experiment 1 and 2 demographics.

	Experiment 1		Experiment 2	
	Mean	SD	Mean	SD
*N*	35	-	35	-
Age	20.54	1.87	20.31	1.86
BFI-10 Extraversion	3.47	0.94	3.19	0.87
BFI-10 Agreeableness	3.17	0.85	2.86	0.85
BFI-10 Conscientiousness	3.76	0.83	3.64	0.82
BFI-10 Neuroticism	2.76	1.04	3.04	0.82
BFI-10 Openness	3.64	0.87	3.73	0.99
RSES	18.91	4.88	18.69	5.95
MBSRQ SIC	3.27	0.39	3.18	0.41
MBSRQ BOMS	3.56	0.97	3.49	0.97
MBSRQ SPA	3.22	0.87	3.43	0.94
MBSRQ CPA	3.67	0.64	3.43	0.63

BFI-10, Big 5 Inventory-10; RSES, Rosenberg Self-Esteem Scale; MBSRQ, Multidimensional Body-Self Relations Questionnaire; SIC, Subjective Importance of Corporality; BOMS, Behaviors Oriented at Maintaining Shape; SPA, Self-evaluation of Physical Attractiveness; CPA, Care for Physical Aspect.

#### Effects of Face, View and Model on experiment questions

**Figure 2:**
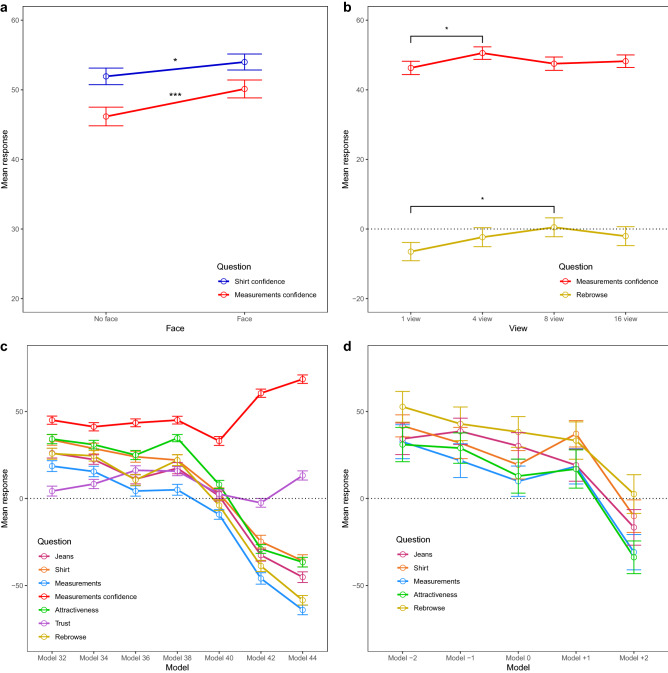
Experiment 1 effects. Mean response for the experiment questions, rated on a scale from − 100 to + 100, showing a significant effect of Face (**a**), View (**b**), Model (**c**), and Model adjusted for participants’ jeans size (**d**) in Experiment 1. Error bars represent standard errors of the mean. *p*-values were corrected using false discovery rate correction. *p < .05, ***p < .001. Jeans = *‘How likely do you think it is that these jeans would fit you?’*, Shirt = *‘How likely do you think it is that this t-shirt would fit you?’*, Shirt confidence = *‘How certain are you?’*, Measurements = *‘How likely do you think it is that this model’s measurements correspond to your own?’*, Measurements confidence = *‘How certain are you?’*, Attractiveness = *‘How attractive do you find this model?’*, Trust = *‘How much do you trust this model (her personality)?’*, Rebrowse = *‘How likely do you think it is that you would choose this model as ‘your model’ for online shopping?’*.

**Figure 3:**
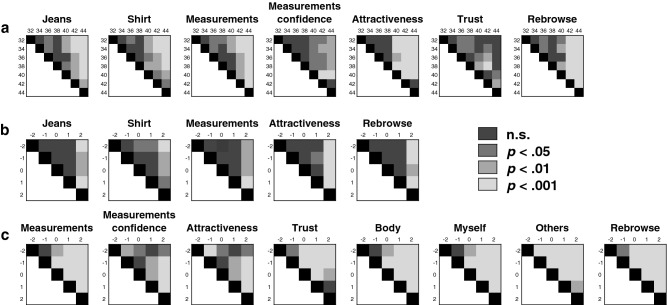
Experiment 1 and 2 Model comparisons. Comparisons for all levels of (**a**) the factor Model in Experiment 1, (**b**) the factor Model adjusted for participants’ jeans size in Experiment 1, and (**c**) the factor Model in Experiment 2. Only questions showing significant effects are presented. The x- and y-axes represent all sizes (levels) of the factor Model. The diagonal is represented using black squares (no comparisons possible), and the part below the diagonal was left blank given the symmetry with the top part. Jeans = *‘How likely do you think it is that these jeans would fit you?’*, Shirt = *‘How likely do you think it is that this t-shirt would fit you?’*, Measurements = *‘How likely do you think it is that this model’s measurements correspond to your own?’*, Measurements confidence = *‘How certain are you?’*, Attractiveness = *‘How attractive do you find this model?’*, Trust = *‘How much do you trust this model (her personality)?’*, Rebrowse = *‘How likely do you think it is that you would choose this model as ‘your model’ for online shopping?’*, Body = *‘I feel as if the body of the model is my own body’*, Myself = *‘The model reflects how I consider myself to be’*, Others = *‘I consider the model to reflect how I want to present myself to others’*.

Normality checks were performed with Shapiro-Wilks tests (all *p*s > 0.103). Data for each question was entered into a 2 × 4 × 7 (Face × View × Model) repeated measures ANOVA and analyzed using R^61^. For analyses using a Bayesian approach, see Supplementary Material. See Table 2 for means and standard deviations for all questions. A significant effect of Face, across all views and models, was observed for the ‘Shirt confidence’ (*F*(1,34) = 4.21, *p* = 0.048, *η*^2^_*p*_ = 0.11) and ‘Measurements confidence’ (*F*(1,34) = 12.58, *p* = 0.001, *η*^2^_*p*_ = 0.27) questions. Participants were more certain about t-shirt fit and measurement correspondence when they were presented with a model with a face compared to a model without a face (see Fig. 2a). For the latter (*F*(3,32) = 3.18, *p* = 0.037, *η*^2^_*p*_ = 0.23) as well as for the ‘Rebrowse’ (*F*(3,32) = 4.75, *p* = 0.008, *η*^2^_*p*_ = 0.31) question, an effect of View was also observed (see Fig. 2b). Follow-up paired samples *t*-tests, corrected for multiple comparisons using false discovery rate (fdr) correction, indicated that for confidence about measurement correspondence, four views were better than one view (*t*(34) = − 3.11, *p* = 0.024, *d* = 0.17). For the ‘Rebrowse’ question, participants gave higher responses when confronted with a model from eight compared to one view (*t*(34) = − 3.42, *p* = 0.012, *d* = 0.29). Finally, all but the ‘Jeans confidence’ and ‘Shirt confidence’ questions showed an effect of Model (‘Jeans’: *F*(6,29) = 7.95, *p* < 0.001, *η*^2^_*p*_ = 0.62; ‘Shirt’: *F*(6,29) = 5.05, *p* = 0.001, *η*^2^_*p*_ = 0.51; ‘Measurements’: *F*(6,29) = 10.42, *p* < 0.001, *η*^2^_*p*_ = 0.68; ‘Measurements confidence’: *F*(6,29) = 6.96, *p* < 0.001, *η*^2^_*p*_ = 0.59; ‘Attractiveness’: *F*(6,29) = 17.04, *p* < 0.001, *η*^2^_*p*_ = 0.78; ‘Trust’: *F*(6,29) = 2.91, *p* = 0.024, *η*^2^_*p*_ = 0.38; ‘Rebrowse’: *F*(6,29) = 17.43, *p* < 0.001, *η*^2^_*p*_ = 0.78). All questions, except the ‘Measurements confidence’ and ‘Trust’ questions, showed higher ratings for the smaller-sized compared to bigger-sized models (see Fig. 2c). See Fig. 3a for comparisons and Supplementary Table S1 for significant comparisons that survived fdr-correction. Adding participants’ jeans size as a between-subject factor into the analysis did not result in significant interactions.

**Table 2:** Mean (SD) for factors Face, View and Model for all self-report measures, rated on a scale from − 100 to + 100, in Experiment 1.

Question	Face		View				Model						
	No face	Face	1	4	8	16	32	34	36	38	40	42	44
Jeans	0.47 (3.77)	− 0.29 (3.69)	− 1.50 (4.12)	1.67 (3.81)	1.21 (3.76)	− 1.00 (3.64)	26.09 (7.04)	22.39 (6.56)	11.14 (6.31)	17.40 (8.25)	1.42 (6.13)	− 32.59 (8.73)	− 45.20 (8.00)
Jeans confidence	44.29 (3.94)	45.97 (4.07)	42.17 (4.38)	46.17 (4.06)	46.88 (3.78)	45.30 (4.07)	46.25 (4.61)	41.25 (4.29)	41.28 (4.76)	48.16 (4.60)	34.20 (4.85)	50.30 (6.14)	54.47 (6.58)
Shirt	6.97 (5.63)	7.53 (5.40)	6.79 (5.33)	7.05 (5.84)	8.70 (5.46)	6.45 (5.70)	33.67 (8.06)	28.73 (8.06)	23.99 (8.05)	22.08 (7.75)	2.90 (7.99)	− 24.81 (9.91)	− 35.82 (9.15)
Shirt confidence	51.93 (3.80)	53.99 (3.88)	50.60 (4.08)	54.33 (3.70)	53.13 (3.91)	53.80 (3.95)	53.07 (4.57)	54.81 (3.96)	51.67 (4.70)	51.29 (4.42)	43.06 (5.04)	56.70 (5.04)	60.13 (5.66)
Measurements	− 11.33 (3.15)	− 10.32 (3.06)	− 12.67 (3.05)	− 10.95 (3.34)	− 9.67 (2.91)	− 10.01 (3.25)	18.61 (7.97)	15.52 (7.36)	4.31 (7.53)	5.01 (7.73)	− 9.19 (6.60)	− 45.96 (8.65)	− 64.06 (7.20)
Measurements confidence	46.16 (4.21)	50.12 (4.30)	46.29 (4.53)	50.55 (3.99)	47.51 (4.48)	48.22 (4.25)	45.04 (5.26)	41.23 (5.15)	43.51 (5.30)	45.05 (4.89)	33.06 (5.48)	60.52 (5.81)	68.58 (6.09)
Attractiveness	11.00 (3.96)	8.21 (4.37)	7.48 (4.38)	9.46 (4.09)	9.85 (4.15)	11.63 (4.15)	34.17 (6.38)	30.98 (5.33)	24.98 (6.33)	34.70 (4.19)	8.03 (4.93)	− 29.02 (6.00)	− 36.61 (7.15)
Trust	6.07 (6.40)	10.44 (5.85)	8.72 (6.09)	7.43 (5.95)	8.24 (5.98)	8.61 (6.11)	4.26 (7.24)	8.33 (6.33)	16.18 (6.27)	15.73 (6.24)	2.53 (6.28)	− 2.50 (6.50)	13.23 (6.53)
Rebrowse	− 0.65 (4.39)	− 4.57 (4.23)	− 6.51 (4.24)	− 2.36 (4.24)	0.49 (3.99)	− 2.06 (4.60)	25.76 (7.77)	24.53 (6.54)	10.64 (7.72)	21.98 (7.11)	− 3.95 (6.93)	− 38.76 (8.05)	− 58.48 (7.04)

Jeans = *‘How likely do you think it is that these jeans would fit you?’*, Jeans confidence = *‘How certain are you?’*, Shirt = *‘How likely do you think it is that this t-shirt would fit you?’*, Shirt confidence = *‘How certain are you?’*, Measurements = *‘How likely do you think it is that this model’s measurements correspond to your own?’*, Measurements confidence = *‘How certain are you?’*, Attractiveness = *‘How attractive do you find this model?’*, Trust = *‘How much do you trust this model (her personality)?’*, Rebrowse = *‘How likely do you think it is that you would choose this model as ‘your model’ for online shopping?’.*

#### Additional analyses of the effect of Model on experiment questions

Given that participants generally seemed to give higher ratings to smaller- in comparison to bigger-sized models irrespective of their own size, we decided to run additional analyses to verify whether we were able to replicate this effect of Model when taking into account the match between participants’ and models’ sizes. As such, we analyzed a subset of our participants (*n* = 19, sizes 36–40) for whom we were be able to identify five different models ranging from size − 2 (two sizes smaller than own size) and − 1 (one size smaller) to 0 (corresponding to own size), + 1 (one size bigger than own size) and + 2 (two sizes bigger). For example, if the participant was a size 38, the model with size 38 became model 0, while the models with sizes 34, 36, 40 and 42 became models − 2, − 1, + 1 and + 2 respectively.

A 2 × 4 × 5 (Face × View × Model) repeated measures analysis revealed a significant effect of Model for the ‘Jeans’ (*F*(4,15) = 7.17, *p* = 0.002, *η*^2^_*p*_ = 0.66), ‘Shirt’ (*F*(4,15) = 5.26, *p* = 0.007, *η*^2^_*p*_ = 0.58), ‘Measurements’ (*F*(4,15) = 5.23, *p* = 0.008, *η*^2^_*p*_ = 0.58), ‘Attractiveness’ (*F*(4,15) = 7.32, *p* = 0.002, *η*^2^_*p*_ = 0.66), and ‘Rebrowse’ (*F*(4,15) = 7.62, *p* = 0.001, *η*^2^_*p*_ = 0.67) questions. For all questions, bigger-sized models again received significantly lower ratings (see Figs. 2d and 3b; see Supplementary Table S2 for significant comparisons that survived fdr-correction). Similar effects of Model (but no interaction effects) were observed when calculating the match between participants’ and models’ sizes using body mass index (BMI) and waist-to-hip ratio (WHR).

#### Post-experiment questions

During the post-experiment questionnaire, where participants were able to compare the models side by side, nine participants were able to correctly identify the model that was physically most similar to themselves, while 12/14 participants chose models that were one to six sizes smaller/bigger respectively. These differences were not significant (*χ*^*2*^(2) = 1.09, *p* = 0.581). A similar result was observed for the question concerning which model participants would choose as their model for online shopping (smaller size = 10, own size = 8, bigger size = 17; *χ*^*2*^(2) = 3.83, *p* = 0.147).

#### Correlations with questionnaires

**Figure 4:**
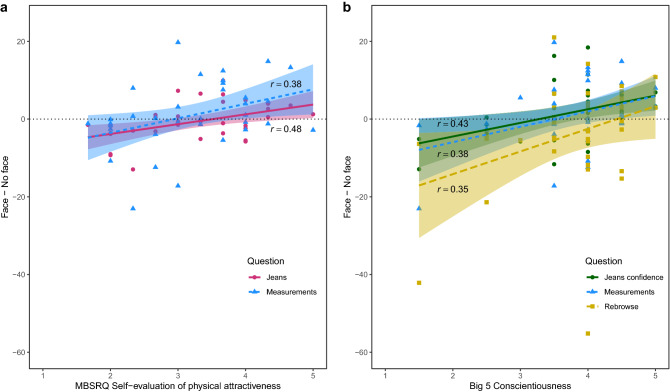
Experiment 1 correlations. (**a**) Correlations between the Self-evaluation of physical attractiveness factor of the Multi-Dimensional Body Self-Relations Questionnaire (MBSRQ) and experiment questions, rated on a scale from − 100 to + 100, of Experiment 1 for the effect of Face. (**b**) Correlations between the Conscientiousness subscale of the Big 5 and experiment questions, rated on a scale from − 100 to + 100, of Experiment 1 for the effect of Face. Shaded areas represent 95% confidence intervals. Individual data points represent raw data. Jeans = *‘How likely do you think it is that these jeans would fit you?’*, Jeans confidence = *‘How certain are you?’*, Measurements = *‘How likely do you think it is that this model’s measurements correspond to your own?’*, Rebrowse = *‘How likely do you think it is that you would choose this model as ‘your model’ for online shopping?’*.

To reduce the number of multiple comparisons, correlations with questionnaire scores were only calculated for the main effects. Fdr-corrected *p*-values are presented for correlations that survived correction. For the ‘Jeans’ and ‘Measurements’ questions, a positive correlation was observed between the effect of Face (Face–No face) and the Self-evaluation of physical attractiveness factor of the MBSRQ (*r* = 0.48, *p* = 0.006 and *r* = 0.38, *p* = 0.026 respectively; see Fig. 4a), suggesting that participants who more positively evaluated their own physical appearance gave higher ratings when observing a model with a face (compared to without a face) when presented with questions about jeans fit and measurements correspondence. Furthermore, for the ‘Jeans confidence’ (*r* = 0.43, *p* = 0.009), ‘Measurements’ (*r* = 0.38, *p* = 0.023), and ‘Rebrowse’ (*r* = 0.35, *p* = 0.042) questions, a significant correlation was observed between the effect of Face and the Conscientiousness scale of the Big 5 (see Fig. 4b), indicating that higher conscientiousness within this participant group was related to higher response for a model with compared to without a face for these three questions.

### Experiment 2

#### Participant characteristics

As in Experiment 1, jeans size was calculated using participants’ measurements. There were 3 participants with jeans size 32, 6 with size 34, 11 with size 36, 9 with size 38, 5 with size 40, 1 with size 42 and 0 with size 44 (similar to the Spanish female distribution^55^).

20 participants indicated to have bought an item online in the past year, with five participants purchasing online with a frequency of one item per week or more. 11 participants indicated to have returned one or more items, mainly due to bad fit. Demographic and questionnaire data is represented in Table 1. Similar to Experiment 1, participants did not fall within the normal ranges for Big 5 Agreeableness (relatively low)^57^, and MBSRQ Subjective importance of corporality (relatively high), Behaviors oriented at maintaining shape (relatively high), Self-evaluation of physical attractiveness (relatively low), and Care for physical aspect (relatively low)^59^.

One participant’s data was not recorded during one trial of the experiment due to technical failure (Model − 2, Face, 4 view).

#### Effects of Face, View and Model on experiment questions

**Figure 5:**
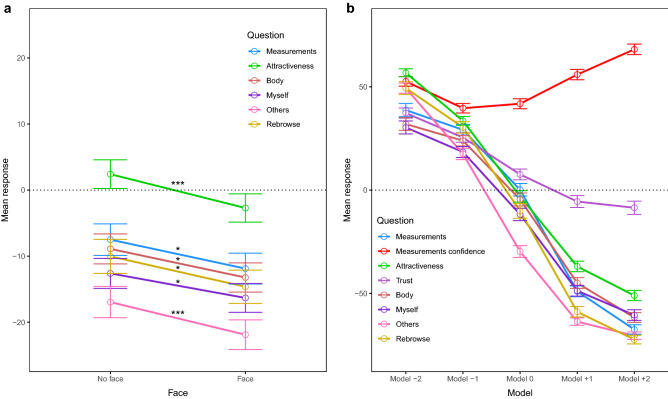
Experiment 2 effects. Mean response for the experiment questions, rated on a scale from − 100 to + 100, showing a significant effect of Face (**a**) and Model (**b**) in Experiment 2. Error bars represent standard errors of the mean. *p* values were corrected using false discovery rate correction. *p < .05, ***p < .001. Measurements = *‘How likely do you think it is that this model’s measurements correspond to your own?’*, Measurements confidence = *‘How certain are you?’*, Attractiveness = *‘How attractive do you find this model?’*, Trust = *‘How much do you trust this model (her personality)?’*, Body = *‘I feel as if the body of the model is my own body’*, Myself = *‘The model reflects how I consider myself to be’*, Others = *‘I consider the model to reflect how I want to present myself to others’,* Rebrowse = *‘How likely do you think it is that you would choose this model as ‘your model’ for online shopping?’*.

Shapiro-Wilks tests indicated that the data was distributed normally (all *p*s > 0.216). Subsequently, data was entered into a 2 × 4 × 5 (Face × View × Model) repeated measures ANOVA, separately for each post-trial question. See Table 3 for an overview of means and standard deviations for all questions. A significant effect of Face was observed for the ‘Measurements’ (*F*(1,34) = 6.08, *p* = 0.019, *η*^2^_*p*_ = 0.15), ‘Attractiveness’ (*F*(1,34) = 19.26, *p* < 0.001, *η*^2^_*p*_ = 0.36), ‘Body’ (*F*(1,34) = 7.02, *p* = 0.012, *η*^2^_*p*_ = 0.17), ‘Myself’ (*F*(1,34) = 4.41, *p* = 0.043, *η*^2^_*p*_ = 0.12), ‘Others’ (*F*(1,34) = 15.55, *p* < 0.001, *η*^2^_*p*_ = 0.31), and ‘Rebrowse’ (*F*(1,34) = 5.03, *p* = 0.032, *η*^2^_*p*_ = 0.13) questions. For all questions, participants gave higher ratings when presented with a model without a face compared to a model with a face (see Fig. 5a). Furthermore, all questions showed a significant effect of Model (‘Measurements’: *F*(4,31) = 52.59, *p* < 0.001, *η*^2^_*p*_ = 0.87; ‘Measurements confidence’: *F*(4,31) = 10.19, *p* < 0.001, *η*^2^_*p*_ = 0.57; ‘Attractiveness’: *F*(4,31) = 58.34, *p* < 0.001, *η*^2^_*p*_ = 0.88; ‘Trust’: *F*(4,31) = 7.54, *p* < 0.001, *η*^2^_*p*_ = 0.49; ‘Body’: *F*(4,31) = 40.11, *p* < 0.001, *η*^2^_*p*_ = 0.84; ‘Myself’: *F*(4,31) = 21.31, *p* < 0.001, *η*^2^_*p*_ = 0.73; ‘Others’: *F*(4,31) = 88.39, *p* < 0.001, *η*^2^_*p*_ = 0.92; ‘Rebrowse’: *F*(4,31) = 95.14, *p* < 0.001, *η*^2^_*p*_ = 0.93). Overall, all but the ‘Measurements confidence’ question resulted in higher ratings for smaller- compared to bigger-sized models (see Fig. 5b). See Fig. 3c for comparisons and Supplementary Table S3 for significant comparisons that survived fdr-correction. No effects of View or interaction effects were observed. Furthermore, adding participants’ jeans size as a between-subject factor into the analysis did not result in significant interactions.

**Table 3:** Mean (SD) for factors Face, View and Model for all self-report measures, rated on a scale from − 100 to + 100, in Experiment 2.

Question	Face		View				Model				
	No face	Face	1	4	8	16	− 2	− 1	0	+ 1	+ 2
Measurements	-7.50 (2.85)	11.88 (2.75)	− 8.36 (3.34)	− 9.48 (2.91)	− 11.28 (2.90)	− 9.66 (3.09)	38.75 (7.68)	29.19 (4.85)	0.15 (6.24)	− 49.03 (6.01)	− 67.52 (4.88)
Measurements confidence	51.87 (4.28)	51.44 (4.23)	48.30 (4.73)	53.78 (4.39)	52.04 (4.27)	52.48 (4.27)	52.65 (51.19)	39.66 (4.75)	41.82 (4.68)	55.95 (5.49)	68.18 (5.34)
Attractiveness	2.41 (4.20)	− 2.71 (4.31)	0.42 (4.54)	0.25 (4.33)	− 1.76 (4.17)	0.50 (4.46)	56.82 (3.66)	33.55 (4.19)	− 3.07 (6.25)	− 36.97 (6.36)	− 51.07 (6.23)
Trust	11.61 (5.19)	10.90 (5.43)	10.63 (5.51)	10.62 (5.35)	10.77 (5.17)	13.00 (5.47)	37.31 (5.90)	25.47 (5.46)	7.60 (6.04)	− 5.54 (7.00)	− 8.55 (7.63)
Body	− 13.23 (2.75)	− 8.90 (2.96)	− 10.52 (3.16)	− 11.51 (2.95)	− 11.38 (3.19)	− 10.84 (3.15)	32.01 (7.20)	23.92 (4.77)	− 4.54 (6.78)	− 45.00 (6.27)	− 61.70 (5.88)
Myself	− 12.61 (2.98)	− 16.32 (2.92)	− 13.54 (3.23)	− 15.93 (3.30)	− 15.58 (2.81)	− 12.82 (3.22)	30.33 (7.28)	18.43 (4.73)	− 11.79 (6.22)	− 48.75 (6.25)	− 60.55 (6.48)
Others	− 16.95 (3.09)	− 21.89 (3.32)	− 19.98 (3.79)	− 20.00 (3.38)	− 19.74 (3.23)	− 17.96 (3.47)	49.35 (4.32)	17.52 (5.48)	− 29.75 (6.31)	− 63.68 (4.06)	− 70.55 (3.75)
Rebrowse	− 10.03 (3.70)	− 14.63 (3.53)	− 12.64 (3.87)	− 13.07 (3.71)	− 14.15 (3.81)	− 9.47 (3.93)	49.40 (6.97)	30.37 (4.98)	− 10.10 (7.74)	− 58.94 (5.60)	− 72.40 (4.85)

Measurements = *‘How likely do you think it is that this model’s measurements correspond to your own?’*, Measurements confidence = *‘How certain are you?’*, Attractiveness = *‘How attractive do you find this model?’*, Trust = *‘How much do you trust this model (her personality)?’*, Body = *‘I feel as if the body of the model is my own body’*, Myself = *‘The model reflects how I consider myself to be’*, Others = *‘I consider the model to reflect how I want to present myself to others’*, Rebrowse = *‘How likely do you think it is that you would choose this model as ‘your model’ for online shopping?’.*

#### Post-experiment questions

When asked which model participants thought was physically most similar to themselves, the majority of participants chose a model that had a bigger size (one–two sizes bigger; 28), compared to a model that had their exact measurements (6), or a smaller size (one–two sizes smaller; 1; *χ*^*2*^(2) = 35.37, *p* < 0.001). A similar result was observed when participants were asked which model they would choose as their model for online shopping (bigger size = 29, own size = 4, smaller size = 2; *χ*^*2*^(2) = 38.80, *p* < 0.001).

#### Correlations with questionnaires

**Figure 6:**
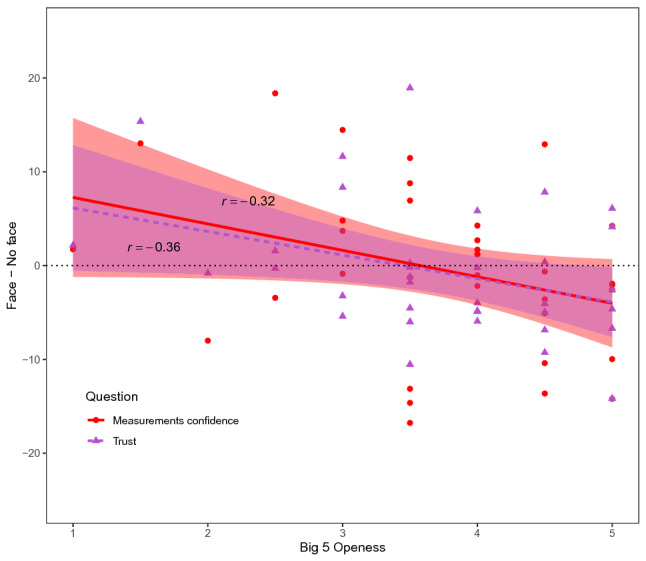
Experiment 2 correlations. Correlations between the Openness subscale of the Big 5 and experiment questions, rated on a scale from − 100 to + 100, of Experiment 2 for the effect of Face. Shaded areas represent 95% confidence intervals. Individual data points represent raw data. Measurements confidence = *‘How certain are you?’* (in response to *‘How likely do you think it is that this model’s measurements correspond to your own?’*), Trust = *‘How much do you trust this model?’*.

As described in Experiment 1, we only correlated the questionnaire subscale scores with the main effects of our repeated measures design. Only correlations that survived fdr-correction for multiple comparisons are presented. For the ‘Measurements confidence’ question, a negative correlation was observed between the effect of Face (Face–No face) and the Openness sub-scale of the Big 5 (*r* = − 0.32, *p* = 0.038; see Fig. 6), indicating that participants who were more open to new experiences were more certain about their answer to the question whether they thought the model’s measurements matched their own when the model was presented without a face. A similar correlation was observed for the ‘Trust’ question (*r* = − 0.36, *p* = 0.037; see Fig. 6), suggesting that participants who scored higher on the Big 5 Openness sub-scale trusted a model without a face more than a model with a face.

## Discussion

In two experiments, we investigated the influence of the presence of facial features, amount of viewpoints, and observed model size on the perceived match between observers’ own bodies and those of observed models/avatars, as well as attitudes towards these observed bodies, as quantified by various self-report measures. Overall, results indicated strong evidence for an effect of observed model size on participants’ judgments, irrespective of participants’ own measurements. As described below, this effect showed an unexpected reversal depending on how and when stimuli were presented to participants. Less evidence was found, however, for an influence of the presence of models’ faces or the amount of viewpoints from which the model could be observed, indicating that only one of our three manipulated visual features (model size) had a strong influence on participants’ ability to accurately identify their own body size in observed models.

In Experiment 1, participants randomly observed photos of real-life models of different sizes wearing an identical outfit. Results indicated that higher ratings were given to models presented with a face compared to models without a face when asked about confidence in their responses concerning how well they thought the observed t-shirt would fit them and how well the model’s measurements corresponded to their own. This effect of rating models with a face higher than models without a face was further modulated by participants’ self-evaluation of physical attractiveness (related to negative attitudes towards one’s own body image^59^) and conscientiousness. Furthermore, when asked about measurement correspondence/rebrowse potential (how likely it was that they would use the observed model for future online shopping), participants provided higher ratings when the model was presented from four/eight different viewpoints compared to only one viewpoint, with 16 viewpoints providing no additional benefit. Finally, participants gave higher ratings to smaller- compared to bigger-sized models during the experiment, irrespective of their own body measurements for nearly all self-report measures (apparel fit, measurement correspondence and confidence, attractiveness, trust, rebrowse potential). These findings concerning model size selection were replicated in Experiment 2, where participants were presented with avatar models based on their own body, with or without distortions to these avatars’ body measurements. Furthermore, when at the end of the experiment—where they were able to view and compare all models freely—participants were asked to select one model they thought was physically most similar to themselves and one model they would choose for further online shopping, they selected bigger-sized models more often than smaller-sized models, again irrespective of their own body measurements (this effect was numerically present but not significant in Experiment 1). Finally, contrary to Experiment 1, participants provided higher ratings for questions about measurement correspondence, attractiveness, body acceptance and identification, and rebrowse potential when presented with models without a face, with this effect being stronger for participants who were more open to new experiences.

In both experiments we observed an effect of Face, although in opposite directions. While we expected models without a face to lead to more accurate body size estimation and identification due to a face’s distracting nature^25,26^, this was only true in Experiment 2. One possible explanation for the discrepancy between these results is the degree of realism that differed across experiments. Facial realism of observed avatars remains a current topic of high interest given that non-realistic avatar representations are faster and more economical to produce while requiring less computational resources^62^, with detailed facial features requiring a lot of the computation effort^63^. However, there is debate as to the direction and extent of the effects of realism, with research suggesting that more humanlike avatars can lead to both higher and lower degrees of^54,64^ or even no changes in^62,65^ body ownership, that high levels of realism evoke feelings of eeriness (uncanny valley effect^66^), and that avatar realism has no effect on measures of appeal and attractiveness^67^. Furthermore, a meta-analysis found that humanlike and realistic avatars led to higher subjective measures of positive social interactions than avatars that were less realistic, although the realism and quality of an avatar’s face were less important than its mere presence^68^. Other studies have indicated that while appearance of an avatar is important (e.g. people spend a lot of time customizing their avatars^12^), the effects of avatar realism are dependent on consumers’ personalities^69^ and opinions and emotions^70^. Finally, other research observed a U-shaped modulation, with stronger brain responses to both the most abstract and most realistic compared to medium-stylized faces (which evoked an uncanny valley effect)^63^. In the current study, faces in Experiment 1 were highly realistic (high-resolution photos of real individuals), while faces of the avatars used in Experiment 2 might not have been realistic or abstract enough. These faces were moderately stylized, with recognizable facial features that were nevertheless not identifiable as a real person’s face. This might have led to lower responses to models with a face compared to models without a face, possibly due to an uncanny valley effect induced by the former. Interestingly, the effect of Face in both experiments was related to the personality type of the observers (as measured by the Big 5 Inventory-10) and self-assessed body image (as measured by the MBSRQ). First, individuals who evaluated themselves as physically more attractive gave higher ratings to models with a face in Experiment 1, indicating that these individuals preferred highly realistic faces, possibly to look for attractiveness markers in the observed models^71^ (although it has also been shown that realism is a bad predictor of perceived attractiveness^67^). Second, while participants who were more conscientious gave higher ratings to models with a face in Experiment 1, models with a face received higher ratings in Experiment 2 from individuals who were less open to new experiences. Thus, these findings seem to indicate that individuals who are highly conscientious seem to be more susceptible to highly realistic and detailed facial features (similar to the effect of realism being stronger for the ‘realist’ personality type^69^), while individuals who score low on openness prefer medium-stylized faces that might induce the uncanny valley effect over the absence of facial features. The effect of individual differences on the uncanny valley effect has largely been neglected, although it has also been found that individuals with a higher need for structure are more prone to experience the effect (seemingly contrary to our results)^72^. Finally, it is important to note, however, that the effect of Face in the current experiments was relatively small. In Experiment 1, only two out of the nine self-report measures showed an effect for this factor. Furthermore, effect sizes—especially compared to the effect sizes of Model—were small in both experiments. This was supported by a Bayesian analysis (see Supplementary Material), which provided substantial evidence to support the null hypothesis that there was no difference between observing models with or without a face. Thus, a more in-depth look at the findings concerning Face suggests that these results should be interpreted with caution.

A similar caution should be upheld when looking at the findings of View. An effect of View was observed only in Experiment 1, and only for self-report of measurement correspondence and rebrowse potential. While these effects were in line with what we predicted (higher ratings when observing a model from more viewpoints, with no additional gain for a full 360° rotation), the effects were small and a Bayesian analysis did not replicate the significant frequentist findings. Thus, while we found some evidence for the idea that viewing a model from more than one angle, without overloading the customer with redundant information, is beneficial for online shopping experiences, further research is necessary.

As predicted, a strong effect of Model was observed. Participants were generally unable to identify the model that best corresponded to their own measurements, nor did they find models with the highest measurement correspondence the most attractive or trustworthy. This inability of individuals to correctly determine own body measurements has been observed repeatedly^2,3,5–8^. Recently, for example, a novel and implicit method was developed to visually depict the internal mental representation of individuals’ own bodies^7^. While they observed that facial self-portraits were relatively accurate, body-shape portraits showed no direct relationships with individuals’ actual body shape, suggesting that offline self-body representations might carry less identity-specific information than faces^73^, or that there might be less genuine inter-individual variability in young female adults’ body shapes. Furthermore, it was found that people have distorted perceptions of relative body proportions both when observing own and others’ bodies, especially when this other body belongs to a person from the same gender^5^. We did not replicate, however, previous research showing that perceptual body estimation is affected by (dis)satisfaction with certain body parts^47,74^, although our measure of these attitudes was relatively limited. In Experiment 2 (and to a lesser extent in Experiment 1), an unexpected discrepancy was observed for self-report in response to two different ways of presenting the model stimuli at different times during the experimental procedure. While smaller-sized models were rated higher during the experiment, when models were randomly presented one at a time, bigger-sized models received higher ratings when all models were presented simultaneously after the experiment. Importantly, both effects were independent from participants’ actual body size (in contrast to previous results^75^). The choice for bigger-sized models post-experiment seems in accordance with research showing that individuals overestimate body size, shape, and proportions^2–6,43^, although higher ratings for smaller-sized models during the experiment are reminiscent of body size underestimation^44–49^ and studies showing that both males and females underestimate their body weight on a virtual body seen from a third-person perspective^76,77^. Other studies have suggested, however, that distortions in perceived body shape and size are dependent on several factors. It has been observed, for example, that distortions in bodily self-representations are highly dependent on affective attitudes towards the self (e.g. self-esteem)^7^. Furthermore, studies looking at body-based scaling have shown that observed objects (including avatars) are perceived relative to the size of one’s own body, rescaling optical information against internal body representations that act as a perceptual reference^78,79^. In other words, when individuals perceive their body as large, observed objects are perceived as smaller and vice versa. The current findings raise the question whether there were differences during and post-experiment that could have induced a similar modulation of over- versus underestimation of body size and have influenced participants’ attitudes. During the experiment, for example, participants observed the models for a pre-defined, limited amount of time (25,500 ms). Research has shown that stimulus duration of bodily information influences local (detailed representation of the body and its parts induced by long stimulus durations) versus global (configural, global representation of the whole body when presented with short stimulus durations) elaboration strategies^80,81^. Although we assumed that 25,500 ms (which is a relatively long duration) was more than enough time to observe the model fully and allow for a local elaboration strategy, it is still possible that post-experiment, where participants had all the time they deemed necessary, they were able to form even fuller, more detailed internal body representations that were used to rate the observed models. As a result, issues such as social desirability and stereotyping could have been more prominent during such a post-experimental phase. Additionally, when participants had the opportunity to compare all models freely, differences between the different models could have become more salient, thus influencing participants’ responses. Finally, it has been suggested that repeated exposure to different body types can lead to adaptation after-effects^82,83^. When selectively presented with thin/fat bodies, individuals will perceive bodies that were previously considered as thinner/fatter as fatter/thinner after adaptation. Furthermore, research has also indicated that perceptual experience with rounder bodies (but not thinner bodies) leads to a higher esthetic appreciation of similar bodies^84^, and that exposure to round models in anorexia nervosa patients alters the perception of their own and others’ bodies^85^. While participants were exposed to an equal amount of thinner/fatter bodies in the current experiment, selective attention to one body type during the experiment could have led to adaptation effects for post-experimental self-report. However, replication of our findings, additionally measuring and/or manipulating internal body representations in both explicit and implicit ways^86^ as well as attention allocation, is necessary. Research using eye-tracking measures has shown, for example, that there is a clear dissociation between fixation location and the location of the regions of the body that are diagnostic for self-estimates of body size (edges of the torso and position of the upper thigh gap)^87^. Finally, it is important to note that the match between own and observed size was dependent on our choice of size classification (based on waist and hips circumvention to determine jeans size) for Experiment 1 (contrary to Experiment 2, where a perfect match was guaranteed for the unadjusted avatar model, since this model was based on a scan of participants’ actual bodies). Our results indicated, however, that the higher ratings for smaller-sized models were similar when adjusting for the match between participants’ and models’ sizes based on jeans size, BMI, or WHR. This is in line with the previously reported underestimation of own body size using BMI measures^44,47–49^ (although other research showed accurate estimation of own body size when observed body shapes were manipulated by continuous changes in BMI^88^).

The current study aimed to systematically identify basic visual features that influence individuals’ perceived match between their own body image and observed models’ bodies, and attitudes towards these models in online environments. Out of three manipulated visual features (facial presence, viewpoints, and model size), only model size showed a strong influence on participants’ performance and ratings. These results shed further insight into the way we perceive and represent our own body, showing that people are generally unable to accurately identify their own body size/shape (replicating previous research) and that facial presence and amount of viewpoints have little influence on this ability. Furthermore, our results suggested that the mode of stimulus presentation might affect the direction of this distorted estimation. By linking these findings to an online retail context, we aimed to advance the understanding of current problems and possible solutions related to erroneous fit and body size identification judgments that are troubling online apparel retail. Future research, developing implicit measures of body identification of and/or attitudes towards observed models/avatars, investigating the link between explicit and implicit measures and overt behavior, manipulating stimulus durations to look at the effects of local versus global elaboration visual strategies, and exploring gender and cultural differences is required to shed more light onto some of these issues. Finally, aside from the MBSRQ, no additional measures of pathological and/or negative body image were included (e.g. Eating Disorder Examination Questionnaire^89^, Eating Disorder Inventory-3^90^), nor did we exclude participants with a current or previous history of eating or body dysmorphic disorders. Given that these disorders greatly impact body size estimation^48,88^, their influence on the effects in the current study are unknown and should be further investigated.

## Methods

### Experiment 1

#### Participants

Sample size was determined following a Bayesian approach using JASP^91^. We scheduled 25 participants, planning to check the Bayes Factor (BF; prior based on a Cauchy distribution, default scale of 0.707, zero-centered) after data collection for this group of participants and—if the stopping criterion had not been reached—after every additional five participants until a maximum of 35 participants. We aimed to stop the experiment whenever the BF reached the threshold for moderate evidence to either support (BF_10_ < 1/6) or reject the null hypothesis (BF_10_ > 6), looking at the factors Face, View, and Model for the ‘Jeans’, ‘Measurements’, and ‘Rebrowse’ questions (see Procedure). Four of the initially scheduled 25 participants did not show up, forcing us to check the BF after data collection for 21, 26, 31, and 35 participants.

35 adults (all female, age in years: range 18–25, *M* = 20.54, *SD* = 1.87) participated in the study in exchange for 12 euros. Participants were recruited through flyers distributed on the university campus, and through university mailing lists. The study was conducted in accordance with the ethical standards laid down in the 1964 Declaration of Helsinki and was granted ethical approval by the local ethics committee at Universidad Carlos III de Madrid (UC3M). All participants provided informed consent beforehand. Additionally, informed consent to publish identifying images in an online open-access publication was obtained from the models depicted in Fig. 1a.

#### Stimuli and apparatus

Stimulus material consisted of images of seven models. Jeans size of different volunteers was determined using a standard European size chart after receiving hips and waist circumvention measurements (used in the aforementioned size chart of the retailer from whom the jeans were purchased). Subsequently, seven different models (all female, age: 18–25 years, height: 170–175 cm) were chosen, each representing a different jeans size (32, 34, 36, 38, 40, 42, 44). We opted to classify our models (and subsequent participants) based on this jeans size (rather than BMI or WHR), given that this best fitted our purpose to explore garment fit perception, although BMI and WHR measures were also obtained. These seven models were invited to a photo shoot, for which they were paid 25 euros. During the photo shoot, models were fitted with the different sizes of an identical pair of blue jeans and white t-shirt (covering all sizes available: XS, S, M, L), as well as a black swimming cap to cover their hair. Any make-up or jewelry was removed. Against a white background, photos of 16 different angles were taken, covering a 360° area in equal steps of 22.5°. Models were asked to take on a neutral facial expression, and stand on a designated spot with their legs slightly apart and their arms at a distance of ± 10 cm removed from the body (see Fig. 1a). During post-processing, images were re-scaled to 953 × 636 pixels. Then, for each image, a corresponding ‘no face’ image was created by pixelating the model’s face so that no facial features could be recognized (using scale-invariant pixelation, i.e. the amount of pixelation is scaled to match the size of the area; www.facepixelizer.com). In total, 224 images from seven different models were obtained.

#### Self-report measures

##### Experiment questions

At the end of each trial, participants were asked nine questions, to be answered on a continuous scale from − 100 to + 100, presented in the following order: *How likely do you think it is that these jeans would fit you?* (‘Jeans’), *How certain are you?* (‘Jeans confidence’), *How likely do you think it is that this t-shirt would fit you?* (‘Shirt’), *How certain are you?* (‘Shirt confidence’), *How likely do you think it is that this model’s measurements correspond to your own?* (‘Measurements’), *How certain are you?* (‘Measurements confidence’), *How attractive do you find this model?* (‘Attractiveness’), *How much do you trust this model (her personality)?* (‘Trust’), *How likely do you think it is that you would choose this model as ‘your model’ for online shopping?* (‘Rebrowse’). Although the focus of Experiment 1 was on accurately determining the model with corresponding jeans size measurements, t-shirt fit was also included for generalizability purposes.

##### Post-experiment questions

At the end of the experiment, participants were presented with images of the different models in a random order and were able to compare them freely side by side. Participants were asked 1) *Which model do you think is physically most similar to you?*, and 2) *Which model would you choose as ‘your model’ for online shopping?*.

#### Questionnaires

##### Online shopping behavior

Participants were asked about their online shopping behavior using questions about the frequency of online website navigation, frequency of purchases, and frequency of returning items. Furthermore, participants indicated the most common reason(s) for the latter.

##### Big 5 Inventory-10 (BFI-10)

The BFI-10^92^, adapted for the Spanish-speaking community^57^, is a short form of the Big 5 Personality test measuring Extraversion, Agreeableness, Conscientiousness, Neuroticism, and Openness using 10 items on a Likert scale from 1 to 5.

##### Rosenberg Self-Esteem Scale (RSES)

The RSES (Spanish version^58^) is a 10-item (Likert scale 1 to 4) self-report questionnaire that measures self-worth by means of both positive and negative feelings about the self.

##### Multidimensional Body-Self Relations Questionnaire (MBSRQ)

The MBSRQ is a well-validated self-report inventory for the assessment of body image. We used a validated 44-item (Likert scale 1 to 5) Spanish version^59^, consisting of four different factors: Subjective importance of corporality, Behaviors oriented at maintaining shape, Self-evaluation of physical attractiveness, and Care for physical aspect. The first and third factor have been shown to be related to negative attitudes towards body image. Furthermore, the former includes five items measuring (dis)satisfaction with different body parts^59^, which were also separately included into the correlation analyses.

#### Design

A 2 × 4 × 7 repeated measures design was used, with Face (Face versus No face), View (1 view versus 4 view versus 8 view versus 16 view) and Model (jeans size 32 versus 34 versus 36 versus 38 versus 40 versus 42 versus 44) as within-subject factors. In the 1 view condition, only one image was shown, corresponding to the 0° viewpoint of the model. In the 4 view condition, a total of five images were shown, which corresponded to viewpoints of the model at 0°, 90°, 180°, 270°, 360°, and 0° (see Fig. 1a). In the 8 view condition, nine images were shown (0°, 45°, 90°, 135°, 180°, 225°, 270°, 315°, and 0°), while in the 16 view condition 17 images were shown, covering the 360° area in equal steps of 22.5°.

#### Procedure

Prior to the start of the experiment, participants’ measurements were obtained, including hips and waist circumvention (to determine jeans size and WHR), and weight and height (to determine BMI). Participants were seated across a computer screen at a distance of ± 65 cm. After participants had been provided with task instructions, they completed a practice phase to ensure they understood the procedure correctly. Presentation software (Version 18.0, Neurobehavioral Systems, Inc.) was used to present the images and record participants’ responses to the experiment questions. Trials were presented in a random order, each with a total duration of 25,500 ms, with each image in the trial being presented for an equal duration. The total trial duration was based on the 16 view condition, where each view was presented for 1,500 ms to allow for sufficient time to observe each image. In all trials, images were presented against a white background. At the end of each trial, the nine experiment questions were presented to participants. After a response to the final question was registered, the first image of the next trial was presented after an inter-trial interval of 500 ms (see Fig. 1a). The experiment comprised of 56 trials. At the end of the experiment, participants completed the post-experiment questions and filled in the questionnaires. The experiment had a maximum total duration of 90 min.

### Experiment 2

#### Participants

Sample size was again determined following a Bayesian approach, using the same stopping criteria as described in Experiment 1. The BF was checked after 20, 25, 30, and 35 participants, looking at the factors Face, View, and Model for the ‘Measurements’, ‘Measurements confidence’ and ‘Rebrowse’ questions (see Procedure).

**Figure 7:**
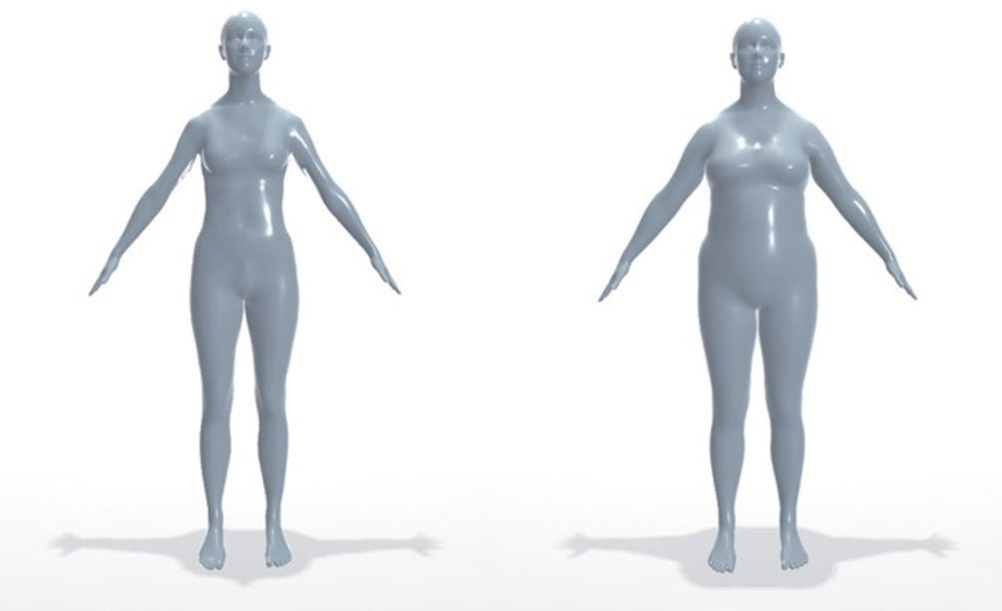
Comparison of parameter 2-induced Model changes in Experiment 2. The Skinned Multi-Person Linear model from one user in Experiment 2, representing the smallest-sized model (Model − 2; left) and biggest-sized model (Model + 2; right). Note that while only parameter 2 (primarily reflecting changes in waist) was changed, this resulted in proportional changes across the entire body.

35 adults (all female, age in years: range 18–25, *M* = 20.31, *SD* = 1.86) participated in the study in exchange for 12 euros. Participants were recruited through flyers distributed on the university campus, and through university mailing lists. The study was conducted in accordance with the ethical standards laid down in the 1964 Declaration of Helsinki and was granted ethical approval by the local ethics committee at UC3M. All participants provided informed consent beforehand, and informed consent to publish identifying images in an online open-access publication was obtained from the participant whose avatar images are used in Figs. 1b and 7.

#### Stimuli and apparatus

Stimulus material consisted of images of a Skinned Multi-Person Linear model (SMPL^93^). We opted for this parametric modeling method given that it is more accurate than previous methods (based on real human data), easier to use for research purposes (fast to render, easy to deploy, compatible with existing rendering engines), and able to ensure similar modeling quality across all users. While specific bodily details might not be captured due to the generalization of this method, it avoids the intensive manual effort (hand rigging a mesh, manually sculpting blend shapes) required by commercial approaches (e.g. computer-generated imagery, CGI). Furthermore, contrary to other research alternatives that focus on learned statistical body models, it is compatible with existing graphics software and rendering engines. After obtaining a 360° full-body capture with a smartphone, the video was post-processed in several steps using custom-made scripts. First, the video was split into 72 images. Then, the front and back images were used to create two mask files to separate background/noise from foreground/person. Using the 72 frames, two mask files, and information about participants’ height and gender, four different files were created^93^: (1) segmentation propagation of the 70 other images based on the manual segmentation of the front and back images, (2) a pointcloud extracted from the segmented images using COLMAP^94,95^; if the segmentation propagation resulted in non-segmented elements, they appeared as outliers in the pointcloud, (3) a clean pointcloud used for optimization, after automatically removing the outliers, and (4) an optimized parametric model that could be visualized in a Unity application developed by the researchers. This visualization allowed us to observe the parametric SMPL model from different angles, and to modify the model’s body shape using ten parameters (reflecting the weight of ten principal components^93^). In the current experiment, we chose to modify the second parameter, which best fitted our purpose given that it primarily represents changes in waist (a primary area of body concern for women^47^). This parameter change resulted in proportional changes across the entire body of the model, including the model’s face (see Fig. 7). For each participant, five different models were obtained by incrementing and reducing this second parameter by 0.5 (reflecting more or less 4 cm in waist circumvention) around the original model. As such, the five different models were labeled as follows: Model − 2 (parameter 2 adjusted by subtracting − 1 from the original value), Model − 1 (subtracting − 0.5), Model 0 (original value), Model + 1 (adding 0.5), and Model + 2 (adding 1). Finally, images (516 × 782 pixels) were obtained from these models as described in Experiment 1 (16 images at 22.5° intervals). Additionally, a corresponding set of ‘no face’ images was created. Given that we had the impression that the pixelized face in Experiment 1 induced unnecessary confusion and to allow us to more clearly distinguish between models with and without a face, we decided to adjust the ‘no face’ condition by covering the face of the models with a white rectangle to completely remove the models’ faces (see Fig. 1b).

#### Self-report measures

##### Experiment questions

Similarly to Experiment 1, participants were presented with eight questions (adapted from Experiment 1 and previous research^53,54^) to be answered on a continuous scale from − 100 to + 100 in a specific order: *How likely do you think it is that this model’s measurements correspond to your own?* (‘Measurements’), *How certain are you?* (‘Measurements confidence’), *How attractive do you find this model?* (‘Attractiveness’), *How much do you trust this model (her personality)?* (‘Trust’), *I feel as if the body of the model is my own body* (‘Body’), *The model reflects how I consider myself to be* (‘Myself’), *I consider the model to reflect how I want to present myself to others* (‘Others’), *How likely do you think it is that you would choose this model as ‘your model’ for online shopping?* (‘Rebrowse’).

##### Post-experiment questions

At the end of the experiment, participants were presented with images of all the models in a random order, and had the opportunity to compare them side by side. Subsequently, they were asked (1) *Which model do you think is physically most similar to you?* and (2) *Which model would you choose as ‘your model’ for online shopping?*.

##### Questionnaires

All questionnaires were equal to the ones used in Experiment 1.

#### Design

A 2 × 4 × 5 repeated-measures design was used, with Face (Face versus No face), View (1 view versus 4 view versus 8 view versus 16 view) and Model (size − 2 versus − 1 versus 0 versus + 1 versus + 2) as within-subject factors.

#### Procedure

During a first session, a 360° full-body capture video of participants was taken in a well-lit room with an iPhone 5S. Background items (including wall coverage) were removed from the room as much as possible. Participants were provided with a tight-fitting blouse and trousers, shown to aid in video post-processing due to their colorful patterns, and were asked to remove all jewelry and to tie back their hair if necessary. Similarly to the model poses in Experiment 1, participants were asked to stand with their legs slightly apart and their arms at ± 10 cm removed from their body. The experimenter then circled around the participant at a distance of ± 2 m to ensure that the whole body was captured. The session had a maximum duration of 15 min.

During the second session, participants were seated across a computer screen at a distance of ± 65 cm. Instructions made sure participants understood the procedure correctly. For all trials, presented in a random order, images were presented against a white background for a total duration of 25,500 ms. After each set of images, participants were asked to answer the eight experiment questions. After a response to the final question was registered, the first image of the next trial was presented after an inter-trial interval of 500 ms (see Fig. 1b). Participants were presented with 40 trials in total and answered the post-experiment questions and filled in the questionnaires at the end of the experiment. This second session had a maximum duration of 45 min.

## Supplementary information

Supplementary Information 1.

Supplementary Information 2.

Supplementary Information 3.

Supplementary Information 4.

Supplementary Information 5.

Supplementary Information 6.

Supplementary Information 7.

## Data Availability

All data generated or analyzed during the experiments is available in the Supplementary Material.
