# Author Correction: Perceived match between own and observed models’ bodies: influence of face, viewpoints, and body size

**DOI:** 10.1038/s41598-020-73063-7

**Published:** 2020-10-08

**Authors:** Lize De Coster, Pablo Sánchez-Herrero, Carlos Aliaga, Miguel A. Otaduy, Jorge López-Moreno, Ana Tajadura-Jiménez

**Affiliations:** 1 DEI Interactive Systems Group, Department of Computer Science and Engineering, Universidad Carlos III de Madrid, Avenida de la Universidad 30, 28911 Leganés, Madrid, Spain; 2 Seddi Labs, Madrid, Spain; 3 Modeling and Virtual Reality Group, Department of Computer Science, Universidad Rey Juan Carlos, Madrid, Spain; 4 Present Address: Multimodal Simulation Lab, Department of Computer Science and Architecture, Computer Systems and Languages, Statistics and Operative Investigation, Universidad Rey Juan Carlos, Madrid, Spain

Correction to: *Scientific Reports* 10.1038/s41598-020-70856-8, published online 19 August 2020

The original version of this Article omitted an affiliation for Jorge López-Moreno. The correct affiliations for Jorge López-Moreno are listed below:

Seddi Labs, Madrid, Spain

Present address: Multimodal Simulation Lab, Department of Computer Science and Architecture, Computer Systems and Languages, Statistics and Operative Investigation, Universidad Rey Juan Carlos, Madrid, Spain

This has now been corrected in the HTML and PDF versions of this Article.

