# Supplementary material for: Perceived match between own and observed models’ bodies: influence of face, viewpoints, and body size
Source: Sci Rep. 2020 Aug 19;10:13991. doi: 10.1038/s41598-020-70856-8 (PMC7438501; doi:10.1038/s41598-020-70856-8)
Supplement: Supplementary file 1 — Supplementary Information 1. [file 41598_2020_70856_MOESM1_ESM.pdf]

Perceived match between own and observed models' bodies: influence of face, viewpoints, and body size – Supplementary Material

Lize De Coster, Pablo Sánchez-Herrero, Carlos Aliaga, Miguel A. Otaduy, Jorge López-Moreno, Ana Tajadura-Jiménez

Results

Experiment 1

See Supplementary Table S1 for pairwise comparisons for the factor Model for all questions showing a main effect of Model (see main article). Supplementary Table S2 provides the same information for Model adjusted for participants' jeans size.

Bayesian analyses of the effects of Face, View and Model on experiment questions

In addition to a frequentist analysis, we performed a Bayesian analysis[1] given that results of the former were not always consistent and – when effects were found – the effect sizes were low, especially concerning the effects of Face and View. Furthermore, for the experiment questions that showed null effects using the frequentist analysis, we wanted to test whether these null results could be considered support for the absence of a difference between the various conditions. We ran a repeated measures analysis in JASP[2] to investigate the main effects of Face, View, and Model, as well as their interactions. For each main and interaction effect we obtained the Bayes Factor (BF_{10}) which represents observing the data under the alternative hypothesis (H_1) compared to the null hypothesis (H_0). We employed a threshold of moderate evidence to support ($BF_{10} < 1/6$) or reject ($BF_{10} > 6$) the null hypothesis.

For all but the 'Measurements confidence' and 'Trust' questions, BF_{10} for the effects of Face, View, and their interaction was smaller than 1/6, supporting the null hypothesis that there were no main effects or interactions involving these factors. For the 'Measurements confidence' question, $BF_{10} = 1.57$, which – although not smaller than 1/6 – did not reach the threshold to reject the null hypothesis. For the 'Trust' question, $BF_{10} = 23.10$, rejecting the null hypothesis that there was no effect of Face. As can be observed in Table 2 (main article), participants

indicated to trust a model with a face more than a model without a face. Concerning the effect of Model, all questions showed a BF_{10} larger than 6 (‘Jeans’: $BF_{10} = 1.38e^{+114}$; ‘Jeans confidence’: $BF_{10} = 1.25e^{+11}$; ‘Shirt’: $BF_{10} = 4.04e^{+112}$; ‘Shirt confidence’: $BF_{10} = 9.73e^{+6}$; ‘Measurements’: $BF_{10} = 1.11e^{+132}$; ‘Measurements confidence’: $BF_{10} = 1.52e^{+45}$; ‘Attractiveness’: $BF_{10} = 7.56e^{+205}$; ‘Trust’: $BF_{10} = 3.12e^{+19}$; ‘Rebrowse’: $BF_{10} = 1.17e^{+155}$), rejecting the null hypothesis that there was no effect of Model. Generally, participants gave higher ratings to smaller- compared to bigger-sized models (see Table 2 main article).

Given that the frequentist analysis showed an effect of Face for the ‘Shirt confidence’ and ‘Measurements confidence’ questions and an effect of View for the ‘Measurements confidence’ and ‘Rebrowse’ questions, and that we did not replicate these effects with our Bayesian analysis, we checked whether there was an indication of an effect of these factors (for the specific questions mentioned) in addition to the effect of the factor Model (which showed a clear effect for all questions). More specifically, we compared models including Model and Face/View (Model + Face/Model + View) to models including only Model by dividing the BF_{10} of the former by the BF_{10} of the latter. The remaining BF_{10} for Face and View is reported when higher than 1. For the ‘Measurements confidence’ question, $BF_{10} = 2.42$ for Face, suggesting that there was some evidence for rejecting the null hypothesis. According to Table 2 (main article), models with a face were given higher ratings than models without a face when asked about certainty concerning measurement correspondence.

Experiment 2

See Supplementary Table S3 for pairwise comparisons for the factor Model for all questions showing a main effect of Model (see main article).

Bayesian analyses of the effects of Face, View and Model on experiment questions

Bayesian analysis was performed as described in Experiment 1. All questions, except the 'Attractiveness' and 'Others' questions, showed a $BF_{10} < 1/6$ for the effects of Face, View, and their interaction. For the 'Attractiveness' ($BF_{10} = .31$) and 'Others' ($BF_{10} = .21$) questions, however, BF_{10} did not reach the threshold to reject the null hypothesis. Conversely, a $BF_{10} > 6$ could be observed for all questions for the effect of Model ('Measurements': $BF_{10} = 7.83e^{+189}$; 'Measurements confidence': $BF_{10} = 3.93e^{+24}$; 'Attractiveness': $BF_{10} = 1.82e^{+287}$; 'Trust': $BF_{10} = 4.18e^{+69}$; 'Body': $BF_{10} = 2.59e^{+156}$; 'Myself': $BF_{10} = 1.98e^{+146}$; 'Others': $BF_{10} = 3.44e^{+292}$; 'Rebrowse': $BF_{10} = 3.85e^{+232}$). Table 3 (main article) indicates that participants gave higher ratings to models with smaller sizes compared to models with bigger sizes.

Since there was a strong effect of Face for all questions (except the 'Measurements confidence' and 'Trust' questions) according to the frequentist analysis, we compared models including Model and Face to models including only Model for the six remaining questions (remaining BF_{10} reported when higher than 1). For the 'Attractiveness' ($BF_{10} = 5.30$) and 'Others' ($BF_{10} = 1.75$) questions, there was some evidence for rejecting the null hypothesis. As can be observed in Table 3 of the main article, models without a face were rated higher than models with a face for these questions.

References

1. Wagenmakers, E.-J. *et al.* Bayesian inference for psychology. Part I: Theoretical advantages and practical ramifications. *Psychon. Bull. Rev.* **25**, 35–57 (2018).

2. JASP Team. JASP (Version 0.12.2)[Computer software]. (2020).

Table S1. Pairwise comparisons for factor Model in Experiment 1.

Significant pairwise comparisons for the factor Model in Experiment 1 for all experiment questions showing a main effect of Model, including *t*-values, false discovery rate (fdr)-corrected *p*-values, and Cohen's *d* as a measure of effect size. Questions that are not presented did not show significant differences. Jeans = *'How likely do you think it is that these jeans would fit you?'*, Shirt = *'How likely do you think it is that this t-shirt would fit you?'*, Measurements = *'How likely do you think it is that this model's measurements correspond to your own?'*, Measurements confidence = *'How certain are you?'*, Attractiveness = *'How attractive do you find this model?'*, Trust = *'How much do you trust this model (her personality)?'*, Rebrowse = *'How likely do you think it is that you would choose this model as 'your model' for online shopping?'*

Question	Comparison	*t*-value	fdr-corrected *p*-value	Cohen's *d*
Jeans	Model 32 vs Model 36	2.46	.027	.38
	Model 32 vs Model 40	3.50	.002	.63
	Model 32 vs Model 42	4.20	< .001	1.25
	Model 32 vs Model 44	5.45	< .001	1.60
	Model 34 vs Model 36	2.59	.021	.30
	Model 34 vs Model 40	3.44	.004	.56
	Model 34 vs Model 42	4.31	< .001	1.20
	Model 34 vs Model 44	5.63	< .001	1.56
	Model 36 vs Model 42	3.65	.002	.97
	Model 36 vs Model 44	4.82	< .001	1.32
	Model 38 vs Model 40	2.16	.050	.37
	Model 38 vs Model 42	3.45	.004	1.00
	Model 38 vs Model 44	4.76	< .001	1.30
	Model 40 vs Model 42	3.03	.008	.76
	Model 40 vs Model 44	4.65	< .001	1.11
	Model 42 vs Model 44	3.43	.004	.25
Shirt	Model 32 vs Model 36	2.84	.013	.20
	Model 32 vs Model 40	3.82	.002	.65
	Model 32 vs Model 42	4.04	< .001	1.09
	Model 32 vs Model 44	4.98	< .001	1.36
	Model 34 vs Model 40	3.77	.002	.54
	Model 34 vs Model 42	3.94	< .001	1.00
	Model 34 vs Model 44	4.88	< .001	1.27
	Model 36 vs Model 40	2.66	.017	.44
	Model 36 vs Model 42	3.51	.002	.91
	Model 36 vs Model 44	4.51	< .001	1.17
	Model 38 vs Model 40	2.97	.009	.41
	Model 38 vs Model 42	3.80	.002	.89
	Model 38 vs Model 44	4.73	< .001	1.15
	Model 40 vs Model 42	2.77	.014	.52
	Model 40 vs Model 44	3.77	.002	.76
	Model 42 vs Model 44	2.61	.017	.20
Measurements	Model 32 vs Model 36	2.44	.028	.31
	Model 32 vs Model 38	2.66	.018	.29
	Model 32 vs Model 40	2.99	.008	.64
	Model 32 vs Model 42	4.16	< .001	1.31
	Model 32 vs Model 44	5.81	< .001	1.84
	Model 34 vs Model 36	2.29	.038	.25
	Model 34 vs Model 40	3.17	.005	.60
	Model 34 vs Model 42	4.18	< .001	1.29
	Model 34 vs Model 44	5.89	< .001	1.85
	Model 36 vs Model 42	3.42	.004	1.05
	Model 36 vs Model 44	5.06	< .001	1.57
	Model 38 vs Model 42	3.42	.004	1.05
	Model 38 vs Model 44	5.18	< .001	1.56
	Model 40 vs Model 42	3.40	.004	.81
	Model 40 vs Model 44	5.63	< .001	1.34
	Model 42 vs Model 44	4.64	< .001	.38
Measurements confidence	Model 32 vs Model 40	2.83	.017	.38
	Model 32 vs Model 42	-2.96	.016	.47
	Model 32 vs Model 44	-3.44	.009	.70
	Model 34 vs Model 40	2.29	.042	.26
	Model 34 vs Model 42	-3.17	.009	.59
	Model 34 vs Model 44	-3.81	.007	.82
	Model 36 vs Model 40	2.32	.042	.33

	Model 36 vs Model 42	-2.67	.021	.52
	Model 36 vs Model 44	-3.23	.009	.74
	Model 38 vs Model 40	2.82	.017	.39
	Model 38 vs Model 42	-2.29	.042	.49
	Model 38 vs Model 44	-3.23	.009	.72
	Model 40 vs Model 42	-5.24	< .001	.82
	Model 40 vs Model 44	-6.08	< .001	1.04
Attractiveness	Model 32 vs Model 40	3.99	< .001	.77
	Model 32 vs Model 42	7.26	< .001	1.72
	Model 32 vs Model 44	7.97	< .001	1.77
	Model 34 vs Model 40	5.00	< .001	.76
	Model 34 vs Model 42	8.78	< .001	1.79
	Model 34 vs Model 44	8.79	< .001	1.81
	Model 36 vs Model 40	3.13	.006	.50
	Model 36 vs Model 42	6.92	< .001	1.48
	Model 36 vs Model 44	7.56	< .001	1.54
	Model 38 vs Model 40	5.98	< .001	.99
	Model 38 vs Model 42	9.44	< .001	2.08
	Model 38 vs Model 44	10.93	< .001	2.06
	Model 40 vs Model 42	7.38	< .001	1.14
	Model 40 vs Model 44	6.61	< .001	1.23
Trust	Model 32 vs Model 36	-2.57	.035	.30
	Model 32 vs Model 38	-2.46	.040	.29
	Model 34 vs Model 36	-2.77	.027	.21
	Model 34 vs Model 38	-2.39	.044	.20
	Model 34 vs Model 42	2.97	.018	.29
	Model 36 vs Model 40	3.69	.005	.37
	Model 36 vs Model 42	4.32	< .001	.49
	Model 38 vs Model 40	3.60	.005	.36
	Model 38 vs Model 42	4.32	< .001	.48
	Model 40 vs Model 44	-2.66	.032	.13
	Model 42 vs Model 44	-3.38	.008	.41
Rebrowse	Model 32 vs Model 36	2.43	.026	.33
	Model 32 vs Model 40	2.79	.014	.68
	Model 32 vs Model 42	4.63	< .001	1.38
	Model 32 vs Model 44	7.35	< .001	1.92
	Model 34 vs Model 36	2.56	.021	.33
	Model 34 vs Model 40	3.59	.002	.71
	Model 34 vs Model 42	5.18	< .001	1.46
	Model 34 vs Model 44	8.16	< .001	2.07
	Model 36 vs Model 42	4.11	< .001	1.06
	Model 36 vs Model 44	6.97	< .001	1.58
	Model 38 vs Model 40	3.34	.003	.62
	Model 38 vs Model 42	4.85	< .001	1.35
	Model 38 vs Model 44	7.54	< .001	1.92
	Model 40 vs Model 42	4.12	< .001	.78
	Model 40 vs Model 44	7.04	< .001	1.32
	Model 42 vs Model 44	4.35	< .001	.44

Table S2. Pairwise comparisons for factor Model, adjusted for participants' jeans size, in Experiment 1.

Significant pairwise comparisons for the factor Model in Experiment 1, adjusted for participants' jeans size, for all experiment questions showing a main effect of Model, including *t*-values, false discovery rate (fdr)-corrected *p*-values, and Cohen's *d* as a measure of effect size. Questions that are not presented did not show significant differences. Jeans = *'How likely do you think it is that these jeans would fit you?'*, Shirt = *'How likely do you think it is that this t-shirt would fit you?'*, Measurements = *'How likely do you think it is that this model's measurements correspond to your own?'*, Attractiveness = *'How attractive do you find this model?'*, Rebrowse = *'How likely do you think it is that you would choose this model as 'your model' for online shopping?'*

Question	Comparison	*t*-value	fdr-corrected *p*-value	Cohen's *d*
Jeans	Model -2 vs Model +2	5.16	< .001	1.49
	Model -1 vs Model +2	4.23	.003	1.05
	Model 0 vs Model +2	3.59	.005	.77
	Model +1 vs Model +2	5.43	< .001	1.28
Shirt	Model -2 vs Model 0	3.24	.013	.38
	Model -2 vs Model +1	2.61	.030	.45
	Model -2 vs Model +2	4.56	< .001	1.15
	Model -1 vs Model +2	3.64	.010	.89
	Model 0 vs Model +2	3.46	.010	.82
	Model +1 vs Model +2	2.94	.018	.66
Measurements	Model -2 vs Model +2	4.19	.003	1.46
	Model -1 vs Model +2	4.06	.003	1.22
	Model 0 vs Model +2	3.97	.003	.99
	Model +1 vs Model +2	4.39	< .001	1.12
Attractiveness	Model -2 vs Model +2	5.01	< .001	1.21
	Model -1 vs Model +1	2.64	.034	.53
	Model -1 vs Model +2	5.37	< .001	1.41
	Model 0 vs Model +2	4.78	< .001	1.19
	Model +1 vs Model +2	4.52	< .001	.84
Rebrowse	Model -2 vs Model +2	4.47	< .001	1.54
	Model -1 vs Model +2	5.71	< .001	1.59
	Model 0 vs Model +2	3.69	.005	1.12
	Model +1 vs Model +2	4.65	< .001	1.14

Table S3. Pairwise comparisons for factor Model in Experiment 2.

Significant pairwise comparisons for the factor Model in Experiment 2 for all experiment questions showing a main effect of Model, including *t*-values, false discovery rate (fdr)-corrected *p*-values, and Cohen's *d* as a measure of effect size. Questions that are not presented did not show significant differences. Measurements = *'How likely do you think it is that this model's measurements correspond to your own?'*, Measurements confidence = *'How certain are you?'*, Attractiveness = *'How attractive do you find this model?'*, Trust = *'How much do you trust this model (her personality)?'*, Body = *'I feel as if the body of the model is my own body'*, Myself = *'The model reflects how I consider myself to be'*, Others = *'I consider the model to reflect how I want to present myself to others'*, Rebrowse = *'How likely do you think it is that you would choose this model as 'your model' for online shopping?'*

Question	Comparison	*t*-value	fdr-corrected *p*-value	Cohen's *d*
Measurements	Model -2 vs Model 0	3.14	.004	.93
	Model -2 vs Model +1	7.12	< .001	2.08
	Model -2 vs Model +2	9.30	< .001	2.79
	Model -1 vs Model 0	4.27	< .001	.88
	Model -1 vs Model +1	9.59	< .001	2.42
	Model -1 vs Model +2	11.97	< .001	3.36
	Model 0 vs Model +1	10.30	< .001	1.36
	Model 0 vs Model +2	12.22	< .001	2.04
	Model +1 vs Model +2	4.69	< .001	.57
Measurements confidence	Model -2 vs Model -1	3.39	.004	.44
	Model -2 vs Model 0	2.59	.018	.37
	Model -2 vs Model +2	-2.75	.013	.50
	Model -1 vs Model +1	-3.39	.004	.54
	Model -1 vs Model +2	-5.48	< .001	.95
	Model 0 vs Model +1	-3.00	.008	.47
	Model 0 vs Model +2	-5.06	< .001	.89
	Model +1 vs Model +2	-4.55	< .001	.38
Attractiveness	Model -2 vs Model -1	4.89	< .001	1.00
	Model -2 vs Model 0	8.74	< .001	1.98
	Model -2 vs Model +1	12.54	< .001	3.06
	Model -2 vs Model +2	14.79	< .001	3.57
	Model -1 vs Model 0	9.58	< .001	1.16
	Model -1 vs Model +1	12.22	< .001	2.21
	Model -1 vs Model +2	14.29	< .001	2.70
	Model 0 vs Model +1	8.69	< .001	.91
	Model 0 vs Model +2	10.82	< .001	1.30
	Model +1 vs Model +2	6.11	< .001	.38

Trust	Model -2 vs Model -1	2.62	.014	.35
	Model -2 vs Model 0	4.45	< .001	.84
	Model -2 vs Model +1	5.47	< .001	1.12
	Model -2 vs Model +2	5.67	< .001	1.14
	Model -1 vs Model 0	4.71	< .001	.55
	Model -1 vs Model +1	4.83	< .001	.84
	Model -1 vs Model +2	4.72	< .001	.87
	Model 0 vs Model +1	3.70	.001	.35
	Model 0 vs Model +2	3.27	.003	.41
Body	Model -2 vs Model 0	2.87	.008	.88
	Model -2 vs Model +1	6.16	< .001	.33
	Model -2 vs Model +2	7.83	< .001	2.41
	Model -1 vs Model 0	3.89	< .001	.82
	Model -1 vs Model +1	7.92	< .001	2.09
	Model -1 vs Model +2	9.78	< .001	2.70
	Model 0 vs Model +1	8.01	< .001	1.05
	Model 0 vs Model +2	10.38	< .001	1.52
	Model +1 vs Model +2	5.33	< .001	.46
Myself	Model -2 vs Model 0	3.44	.002	1.05
	Model -2 vs Model +1	6.38	< .001	1.97
	Model -2 vs Model +2	7.22	< .001	2.23
	Model -1 vs Model 0	4.69	< .001	.92
	Model -1 vs Model +1	8.06	< .001	2.05
	Model -1 vs Model +2	8.62	< .001	2.35
	Model 0 vs Model +1	7.41	< .001	1.00
	Model 0 vs Model +2	8.08	< .001	1.30
	Model +1 vs Model +2	3.96	< .001	.31
Others	Model -2 vs Model -1	4.52	< .001	1.09
	Model -2 vs Model 0	9.21	< .001	2.47
	Model -2 vs Model +1	15.90	< .001	4.56
	Model -2 vs Model +2	17.24	< .001	5.01
	Model -1 vs Model 0	12.88	< .001	1.35
	Model -1 vs Model +1	15.25	< .001	2.85
	Model -1 vs Model +2	15.48	< .001	3.17
	Model 0 vs Model +1	7.40	< .001	1.08
	Model 0 vs Model +2	7.95	< .001	1.33
	Model +1 vs Model +2	3.43	.003	.30
Rebrowse	Model -2 vs Model -1	2.48	.018	.53
	Model -2 vs Model 0	4.58	< .001	1.37
	Model -2 vs Model +1	10.17	< .001	2.90
	Model -2 vs Model +2	11.72	< .001	3.43
	Model -1 vs Model 0	6.12	< .001	1.05
	Model -1 vs Model +1	14.32	< .001	2.85
	Model -1 vs Model +2	15.47	< .001	3.54
	Model 0 vs Model +1	9.71	< .001	1.22
	Model 0 vs Model +2	11.55	< .001	1.63
	Model +1 vs Model +2	4.53	< .001	.43
